# Biology: Motion is Function

**DOI:** 10.1093/function/zqac030

**Published:** 2022-06-14

**Authors:** Lauren Gerard Koch, Steven L Britton

**Affiliations:** Department of Physiology & Pharmacology, The University of Toledo, Toledo, OH 43614, USA; Department of Molecular and Integrative Physiology and Department of Anesthesiology, University of Michigan, Ann Arbor, MI 48109, USA

**Keywords:** energy transfer, entropy, quantum biology, thermodynamics, evolution, rats, low capacity runners, high capacity runners

## Abstract

In 1966 Francis Crick declared that: “The ultimate aim of the modern movement in biology is to explain all biology in terms of physics and chemistry.” This motivated us to contemplate approaches that unify biology at a fundamental level. Exploration led us to consider the features of energy, entropy, and motion. Overall, it can be considered that motion of matter is the feature of life function. No motion. No function. In initial work we evaluated the hypothesis that the scope for biologic function is mediated mechanistically by a differential for energy transfer. Maximal treadmill running capacity served as a proxy for energy transfer. The span for capacity was estimated “biologically” by application of two-way artificial selection in rats for running capacity. Consistent with our “Energy Transfer Hypothesis” (ETH), low physical health and dysfunction segregated with low running capacity and high physical health and function segregated with high running capacity. The high energy yield of aerobic metabolism is also consonant with the ETH; that is, amongst the elements of the universe, oxygen is second only to fluorine in electronegativity. Although we deem these energy findings possibly correct, they are based on correlation and do not illuminate function via fundamental principles. For consideration of life, Entropy (2nd Law of thermodynamics) can be viewed as an open system that exchanges energy with the universe operating via nonequilibrium thermodynamics. The Principle of Maximal Entropy Production (MEP) states that: If a source of free energy is present, complex systems can intercept the free energy flow, and self-organize to enhance entropy production. The development of Benard convection cells in a water heat gradient demonstrate simplistic operation of MEP. A direct step forward would be to explain the mechanism of the obligatory motion of molecules for life function. Motion may be mediated by operation of “action at a distance” for molecules as considered by the Einstein-Podolsky-Rosen Paradox and confirmed by JS Bell. Magnetism, electricity, and gravity are also examples of action at a distance. We propose that some variant of “action at a distance” as directed by the property of Maximal Entropy Production (MEP) underwrites biologic motion.

## Initial Idea

The following series led us to formulate a mechanistic view of biology based upon energy transfer.^1^ We had noted in the clinical literature the development of a strong statistical link between low capacity for oxygen metabolism and high risk for disease that started about 40 years ago and is now extensively confirmed in large-scale contemporary studies. Indeed, it is widely accepted that low capacity for oxygen metabolism (aerobic function) is more of a risk factor for death relative to other clinical indicators including type 2 diabetes, smoking, and coronary artery disease. Thus, based upon association studies, it seemed plausible that oxygen metabolism is a unifying common denominator for the distribution of biologic function and health.^2^ From this linkage of complex disease risks with low capacity for oxygen metabolism, we initiated the *Energy Transfer Hypothesis (ETH): Variation in capacity for energy transfer is the central mechanistic determinant of the divide between disease and health*. We adopted the term “energy transfer” to be inclusive of both aerobic and anaerobic metabolism.

## We Devised Two Goals to Guide Progress

### Goal One: Test Directly the Energy Transfer Hypothesis At the Biological Level.

Our association with quantitative geneticist John P. Rapp^3^ led us to consider that artificial selective breeding can be used as a tool to test the ETH. That is, as an unbiased test of the ETH, we reasoned that: *divergent (two-way) selection based on low and high maximal running capacity in rats would yield contrasting models of capacity for energy transfer that also divide for disease risks and biologic dysfunction*.

**Figure 1.**
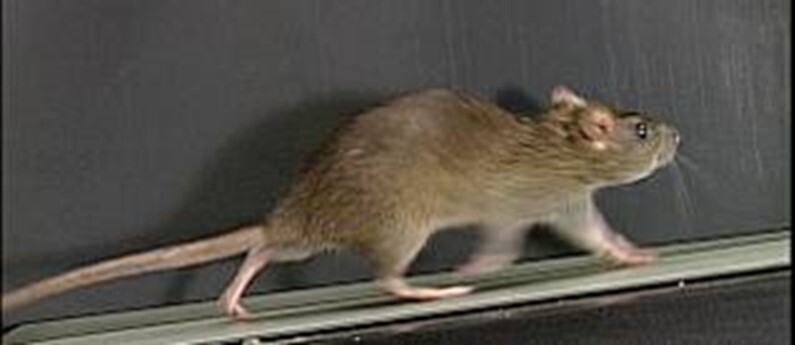
Rats selectively bred for high running capacity (maximal energy transfer) are healthier and live longer than rats selected for low running capacity.

**Figure 2.**
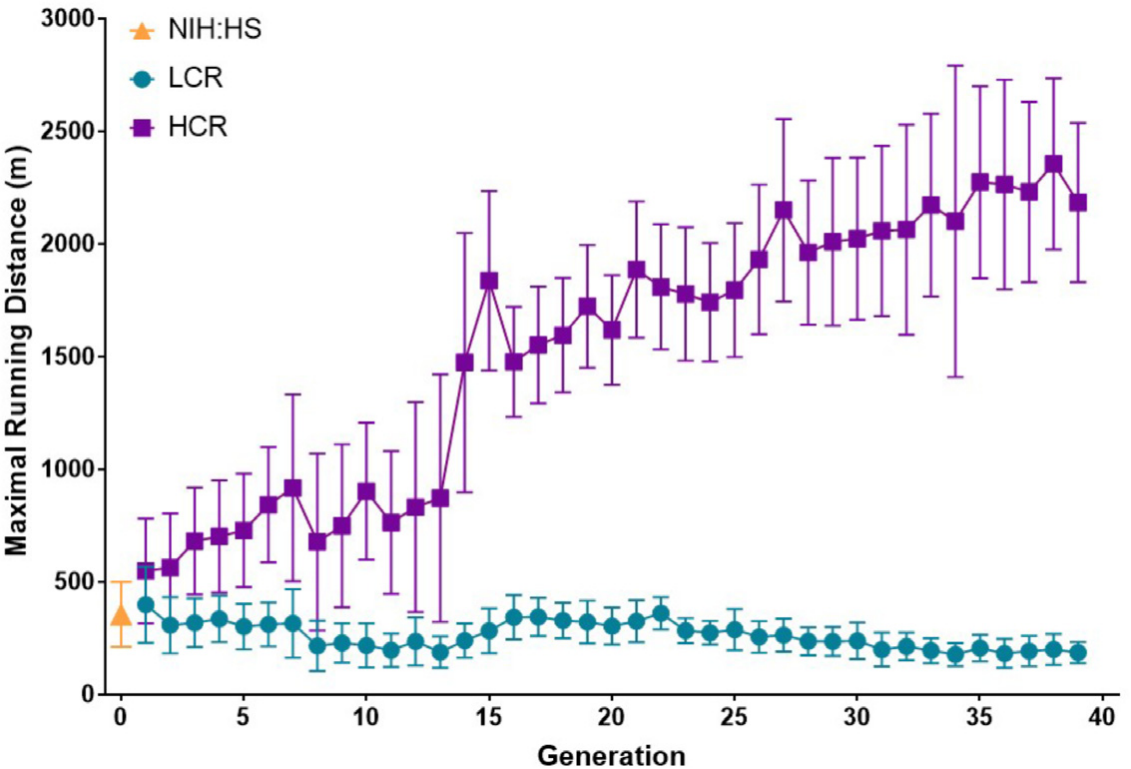
Forty generations of two-way selection for maximal running capacity starting with a founder stock of genetically heterogenous rats (N/NIH:HS). High Capacity Runners (HCR) and Low Capacity Runners (LCR).

In 1996, we started a large-scale selection experiment for low and high intrinsic (i.e., not trained) running capacity. Selection was based upon a test for maximal distance run to exhaustion on a motorized treadmill using a velocity-ramped protocol similar to tests used clinically (Figure 1). As predicted, because we started with a genetically heterogeneous founder population (N: NIH),^4^ the rats responded robustly to selective breeding for low and high running capacity. By generation 40 of selection (18 years), the lines (Low Capacity Runners; LCR vs. High Capacity Runners; HCR) differed by more than 8-fold for maximal running distance (Figure 2). Selection is consistent with the ETH. That is, disease risks segregated with selection for low capacity exercise, and a resilience to risks including increased longevity, segregated with selection for high capacity exercise. Essentially every study using the LCR and HCR rats has revealed a divide for biologic function that also often demonstrated a property of disease. This conclusion was derived from + 125 published studies performed in ^∼^40 institutions in 11 countries.

The strong clustering of disease and dysfunction with low exercise capacity suggests common causality. While it is widely hypothesized that some aspect of mitochondrial dysfunction is the mediator of the disease-exercise connection, a general mechanistic explanation for this association has not been defined. At the applied level, it is not surprising that transfer (i.e., transplantation) of isolated healthy mitochondria into diseased tissues provides therapeutic retrieval.^5^ The strong differential for mitochondrial function between the LCR (low) and HCR (high) rat models provides substrate for exploring the origin and treatment of complex diseases.^6,7^

### Goal Two: Formulate an Explanation of the Energy Transfer Hypothesis.

There is to some degree an unwarranted, intuitive-based, notion that evermore fine-grained biologic information will yield mechanistic insight that can be integrated into quantitative predictive models. Yet, even with detailed information about all molecular processes including intracellular machines, interactions, and regulatory pathways, the formula for building a comprehensive mechanistic model remains elusive. In large part, this is because biological function has an unknown number of degrees of freedom (the number of independent factors required to specify a system: e.g., gene expression, protein levels, etc.) which also can assume an unknown number of physico-chemical states. We sought a path for explanation of operation for the ETH that was objective. Our search led us to a 1981 paper by Hans Krebs and Jack Baldwin^8^ titled “The evolution of metabolic cycles.” From this paper we extracted the critical view that: *within evolution, life evolves along the transfer of energy for motion*.

#### Function via ATP-mediated motion

In 2000, Vale and Milligan^9^ published an influential paper titled “The way things move: looking under the hood of molecular motor proteins.” The tenet is that that kinesin and myosin share a common core structure and convert energy from ATP into protein motion using a similar conformational change strategy. While probably true, this statement is of narrow value without explanation of what property drives the summed behavior of the atoms of molecular motors to manifest the motion of life. Similarly, it is widely accepted that evolution is mediated by natural selection as it is influenced by mutation without accounting for what property produces changes in atoms that mediate mutation.

Some investigators have provided abstract explanatory approaches for biology. Somewhat recently this includes Ilya Prigogine,^10^ Jeremy England,^11^ and Robert Endres.^12^ Endres’ most general thesis proposes that energy dissipation leading to organization seems to be the fundamental property of matter in response to an external energy transfer. That is: (1) Total entropy (S) is always increasing (or is zero at equilibrium., (2) Entropy can temporarily decrease and ordered systems can form (order from disorder), (3) Ordered systems dissipate energy faster than non-ordered systems, and (4) Systems tend towards Maximal Entropy Production (MEP). Simplistically, consider an increment in entropy (dS) as a two-part system:

$$\begin{eqnarray*}
\rm{dS = d_{e}S + d_{i}S}
\end{eqnarray*}
$$

where d_e_S is the flow of entropy due to exchanges with the surroundings (external environment) and d_i_S is the entropy production due to processes inside the system such as diffusion, chemical reactions, and heat conduction (internal metabolism). That is, living systems are dependent on outside energy fluxes (d_e_S) to maintain their organization and dissipate energy gradients to carry out self-organizing processes. The fundamental idea is that the origin and evolution of biological systems are paths for energy transfer via dissipative structures that lead to the development of ordered systems that dispel energy even more effectively (MEP). Per unit mass, living things, such as a grass lawn, most certainly produce more entropy relative to inanimate objects such as rocks.^13^

## Biology Is Difficult At the Mechanistic Level Because of Two Possibly Related Features

### Feature One: The Omnigenic Model

Recent work **of Jonathan Pritchard and colleagues****^14^** provides a new view for the genetic contribution to complex traits as summarized in three statements:

1. The bulk of heritability can be attributed to a huge number of genetic variants, each with a very small effect, that have no “currently obvious” functional connection to phenotype.
2. These variants tend to be spread very broadly across the genome.
3. For complex traits, such as height, analyses demonstrate that as many as half of all SNPs may be in linkage disequilibrium (i.e., associated, inherited together) with causal variants.

Much of the progress in classical genetics has come from detailed molecular work to dissect the biological mechanisms of individual mutations. That work operated on the expectation that there is a relatively direct molecular pathway from genotype to phenotype. Yet, Pritchard demonstrates that the genetic basis of complex traits is highly diffuse. The Omnigenic Model makes it clear that molecular mapping from genotype to phenotype is difficult to conceptualize.

The Omnigenic Model type of function is, however, consistent with our above theoretical arguments that emergence and evolution of life funnel largely through a single mechanistic entropic path. That is, the emergence of life represented pathways for enhanced energy transfer that utilized the entire genome for moment-to-moment infinitesimal adjustments to environment. In refinement, we propose that the small effect phenotypes are driven by variants that mediate energy transfer capacity that is concealed by the miniscule scale of each accumulative genetic change that occurs with evolution. Understanding how the small individual variants aggregate to produce a “map” for phenotypes is a new frontier.

### Feature Two: The mediator of biologic motion is not known

Forces can be divided into two types: (1) those that act by direct contact (“collision”) that is termed *local reality* and, (2) those that act at a distance, where there is no apparent physical contact between the objects termed *action at a distance*. The idea of local reality purports that an event at one location cannot affect what happens at a distance. The principle of local reality was long regarded as a bedrock assumption about the laws of physics and fits with the human intuitive view of reality (13).

In 1935 Einstein, Podolsky, and Rosen^15^ discussed, without resolve, whether quantum mechanics permits action at a distance. Then, in 1964 John Stewart Bell published a paper titled: “On the Einstein Podolsky Rosen Paradox” that enunciated his theorem showing that quantum mechanics allows instantaneous connections for action at a distance.^16^ This paper was published in a short-lived physics journal and has subsequently been used to prove operation of action at a distance many times.^17^ Magnetism, electrical charge, and gravitation are examples of forces that operate via action at a distance. In a speculative step, we propose that some variant of action at a distance drives biologic motion as directed by the property of maximal entropy production. Action at distance is a property as real as blood pressure or an action potential in physiology. Mathematical and machine learning resolution of these features may be required for progress. See: “The Unreasonable Effectiveness of Mathematics in the Natural Sciences" as per Eugene Wigner.^18^

## Funding

The LCR-HCR rat model system was supported by the Office of Research Infrastructure Programs/OD grant ROD012098A (to LGK and SLB) from the National Institutes of Health.

## Data Availability

The data underlying this article will be shared on reasonable request to the corresponding author.

## References

[1] Koch LG, Britton SL. Theoretical and Biological Evaluation of the Link between Low Exercise Capacity and Disease Risk. Cold Spring Harb Perspect Med. a029868, 2018;8(1). Epub 2017/04/09. doi: 10.1101/cshperspect.a029868. PubMed PMID: 28389512; PMCID: *PMC Pending*. 28389512 PMC5749140

[2] Wisloff U, Najjar SM, Ellingsen O, Haram PM, Swoap S, Al-Share Q, Fernstrom M, Rezaei K, Lee SJ, Koch LG, Britton SL. Cardiovascular risk factors emerge after artificial selection for low aerobic capacity. Science. 2005;307(5708):418–420.. Epub 2005/01/22. doi: 10.1126/science.1108177. PubMed PMID: 15662013; PMCID: *PMC Pending*. 15662013

[3] Rapp JP. Genetic analysis of inherited hypertension in the rat. Physiol Rev. 2000;80(1):135–172.. Epub 2000/01/05. doi: 10.1152/physrev.2000.80.1.135. PubMed PMID: 10617767. 10617767

[4] Hansen C, Spuhler K. Development of the National Institutes of Health genetically heterogeneous rat stock. Alcoholism: Clin Experim Res. 1984;8(5):477–479.. Epub 1984/09/01. PubMed PMID: 6391259. 10.1111/j.1530-0277.1984.tb05706.x 6391259

[5] Guariento A, Piekarski BL, Doulamis IP, Blitzer D, Ferraro AM, Harrild DM, Zurakowski D, Del Nido PJ, McCully JD, Emani SM. Autologous mitochondrial transplantation for cardiogenic shock in pediatric patients following ischemia-reperfusion injury. J Thorac Cardiovasc Surg. 2021;162(3):992–1001.. Epub 2020/12/23. doi: 10.1016/j.jtcvs.2020.10.151. PubMed PMID: 33349443. 33349443 10.1016/j.jtcvs.2020.10.151

[6] Seifert EL, Bastianelli M, Aguer C, Moffat C, Estey C, Koch LG, Britton SL, Harper M-E. Intrinsic aerobic capacity correlates with greater inherent mitochondrial oxidative and H2O2 emission capacities without major shifts in myosin heavy chain isoform. J Appl Physiol. 2012;113(10):1624–1634.. doi: 10.1152/japplphysiol.01475.2011. PubMed PMID: WOS:000311208700014. 22995392 PMC3524658

[7] Aon MA, Cortassa S, Juhaszova M, Gonzalez-Reyes JA, Calvo-Rubio M, Villalba JM, Lachance AD, Ziman BD, Mitchell SJ, Murt KN, Axsom JEC, Alfaras I, Britton SL, Koch LG, de Cabo R, Lakatta EG, Sollott SJ. Mitochondrial health is enhanced in rats with higher vs. lower intrinsic exercise capacity and extended lifespan. NPJ Aging Mechan Dis. 2021;7(1):1. doi: 10.1038/s41514-020-00054-3. PubMed PMID: 33398019; PMCID: PMC7782588. PMC7782588 33398019

[8] Baldwin JE, Krebs H. The evolution of metabolic cycles. Nature. 1981;291(5814):381–382.. Epub 1981/06/04. PubMed PMID: 7242661. 7242661 10.1038/291381a0

[9] Vale RD, Milligan RA. The way things move: looking under the hood of molecular motor proteins. Science. 2000;288(5463):88–95.. Epub 2001/02/07. doi: 10.1126/science.288.5463.88. PubMed PMID: 10753125. 10753125

[10] Prigogine I. Time, structure, and fluctuations. Science. 1978;201(4358):777–785.. Epub 1978/09/01. doi: 10.1126/science.201.4358.777. PubMed PMID: 17738519. 17738519

[11] England JL. Statistical physics of self-replication. J Chem Phys. 2013;139(12):121923. Epub 2013/10/05. doi: 10.1063/1.4818538. PubMed PMID: 24089735. 24089735

[12] Endres RG. Entropy production selects nonequilibrium states in multistable systems. Sci Rep. 2017;7(1):14437. doi: 10.1038/s41598-017-14485-8. 29089531 PMC5663838

[13] Whitfield J. Survival of the Likeliest?. PLoS Biol. 2007;5(5):e142. doi: 10.1371/journal.pbio.0050142. 17503967 PMC1868074

[14] Boyle EA, Li YI, Pritchard JK. An Expanded View of Complex Traits: from Polygenic to Omnigenic. Cell. 2017;169(7):1177–1186.. Epub 2017/06/18. doi: 10.1016/j.cell.2017.05.038. PubMed PMID: 28622505; PMCID: PMC5536862. 28622505 PMC5536862

[15] Einstein A, Podolsky B, Rosen N. Can quantum-mechanical description of physical reality be considered complete?. Phys Rev. 1935;47(10):777–780.. doi: 10.1103/PhysRev.47.777. PubMed PMID: WOS:000201519800010.

[16] Bell JS. On the Einstein Podolsky Rosen paradox. Physics Physique Fizika. 1964;1(3):195–200.. doi: 10.1103/PhysicsPhysiqueFizika.1.195.

[17] Salart D, Baas A, Branciard C, Gisin N, Zbinden H. Testing the speed of ‘spooky action at a distance’. Nature. 2008;454(7206):861–864.. Epub 2008/08/16. doi: 10.1038/nature07121. PubMed PMID: 18704081. 18704081

[18] Wigner EP. The unreasonable effectiveness of mathematics in the natural sciences. Richard courant lecture in mathematical sciences delivered at New York University, 1960. Commun Pure Appl Math. 1960;13(1):1–14.. doi: 10.1002/cpa.3160130102.

